# Green synthesis of silk sericin-embedded silver nanoparticles and their antibacterial application against multidrug-resistant pathogens

**DOI:** 10.1186/s43141-021-00176-5

**Published:** 2021-05-17

**Authors:** Md. Abdullah Al Masud, Hamid Shaikh, Md. Shamsul Alam, M. Minnatul Karim, M. Abdul Momin, M. Ariful Islam, G. M. Arifuzzaman Khan

**Affiliations:** 1 Department of Applied Chemistry and Chemical Engineering, Islamic University, Kushtia, 7003 Bangladesh; 2 Department of Chemical Engineering, King Saud University, Riyadh, Saudi Arabia; 3 Department of Biotechnology and Genetic Engineering, Islamic University, Kushtia, 7003 Bangladesh; 4 Department of Electric and Electronic Engineering, Islamic University, Kushtia, 7003 Bangladesh

**Keywords:** Green synthesis, Silk sericin, SS-AgNPs, Antibacterial activity

## Abstract

**Background:**

The green synthesis strategy of metallic nanoparticles (NPs) has become popular due to being environmentally friendly. Stable silver nanoparticles (AgNPs) have been synthesized by natural products such as starch, soy protein, various extract of leaves, barks, and roots functioning both as reducing and stabilizing agents. Likewise, silk sericin (SS) is a globular protein discarded in the silk factory might be used for NP synthesis. In this research, we focus on the green synthesis and stabilization of AgNPs by SS as well as assessment of their antibacterial activities against some drug-resistant pathogen.

**Results:**

SS was extracted from *Bombyx mori* silkworm cocoons in an aqueous medium. 17 w/w% of dry sericin powder with respect to the cocoon’s weight was obtained by freeze-drying. Furthermore, AgNPs conjugated to sericin, i.e., SS-capped silver nanoparticles (SS-AgNPs) were synthesized by easy, cost-effective, and environment-friendly methods. The synthesized SS-AgNPs were characterized by UV-visible spectroscopy, Fourier-transform infrared-attenuated total reflection (FTIR-ATR) spectroscopy, transmission electron microscopy (TEM), and X-ray diffraction measurement. It has been found from the absorbance of UV-visible spectroscopy that a higher percent of SS-AgNPs was obtained at a higher concentration of silver nitrate solution. FTIR-ATR spectra showed that the carboxylate groups obtained from silk sericin act as a reducing agent for the synthesis of silver nanoparticles, while NH_2_+ and COO− act as a stabilizer of AgNPs. The X-ray diffractogram of SS-AgNPs was quite different from AgNO_3_ and sericin due to a change in the crystal structure. The diameter of AgNPs was around 20–70 nm observed using TEM. The synthesized SS-AgNPs exhibited strong antibacterial activity against multidrug-resistant pathogens, *Escherichia coli* and *Pseudomonas aeruginosa*. Minimal inhibitory/bactericidal concentrations against *E. coli* and *P. aeruginosa* were 20μg/mL.

**Conclusions:**

This study encourages the use of *Bombyx mori* for the ecofriendly synthesis of SS-AgNPs to control multidrug-resistant microorganisms.

## Background

By-products contribute substantially towards industrial pollution. However, it is possible to turn by-products into valuable items by using appropriate management systems. Industrial pollution is not always from harmful, toxic ingredients, or heavy metals, but it can also result from ecofriendly products. For example, in the silk industry, water pollution occurs when wastewater from the degumming process runs out through drains. Worldwide, it is estimated that 400,000 tons of dry cocoons produce 50,000 tons of sericin each year. However, sericin is mostly discarded in silk processing wastewater and results in a high chemical oxygen demand (COD) and biological oxygen demand (BOD) levels in the degumming wastewater [1]. Therefore, the recovery and reuse of discarded sericin are beneficial because of its economic, social, and environmental advantages.

Silk sericin is a type of water-soluble globular protein derived from the silkworm *Bombyx mori*, and it represents a family of proteins whose molecular mass ranges from 10 to 310 kDa [2–6]. Silk sericin has many unique properties, including biodegradability, nontoxicity, oxidation resistance, antimicrobial activity, ultraviolet (UV) resistance, and moisture absorption [1, 2, 7]. The physico-chemical properties of molecules are responsible for numerous applications in biomedicine and are influenced by the extraction method and silkworm lineage, which can lead to variations in the molecular weight and amino acid concentration of sericin. The silk sericin has been widely used in biomaterial applications due to its biocompatibility, biodegradability, and anti-oxidative and bioactive activities [8]. The presence of highly hydrophobic amino acids and their antioxidant potential makes it possible for sericin to be applied in the food and cosmetic industry. Silk sericin membranes are good bandage materials, and their film has adequate flexibility and tensile strength. Due to its substantial biocompatibility and infection-resistant nature, it is a novel wound coagulant material. Additionally, its flexibility and water absorption properties enable its use as a smooth cure for defects in the skin and do not cause any peeling of the skin under regeneration when detached from the skin [9]. Zhoarigetu et al. mentioned that sericin can be used as a raw material for making contact lenses: oxygen permeable membranes comprising fibroin and sericin with 10–16% water are used for making contact lenses and as artificial skin [1]. Masahiro et al. reported that the consumption of sericin enhances the bioavailability of Zn, Fe, Mg, and Ca in rats and suggested that sericin is a valuable natural ingredient for the food industry [10]. Recently, sericin has been used as a reducing and stabilizing agent in the synthesis of metal nanoparticles [7]. The primary requirement for the synthesis of metal nanoparticles (NPs) is reducing the biological agents and other constituents present in the cells acting as stabilizing and capping agents, so there is no need to add capping and stabilizing agents from outside [11–15]. He et al. developed a novel, simple, one-step biosynthesis method to prepare a sericin-silver nanoparticle composite in situ in solution. Sericin served as the reductant of silver ion, the dispersant and stabilizer of the prepared sericin-silver nanoparticle composite [16]. Tahir et al. (2020) evaluated the antibacterial activity of sericin-conjugated silver NPs synthesized using sericin as a reducing and capping agent [13, 17]. Aramwit et al. [8] synthesized silk sericin (SS)-capped silver nanoparticles (AgNPs) under alkaline conditions (pH 11) using SS as a reducing and stabilizing agent instead of toxic chemicals. Most relevant studies stated that SS-capped AgNPs have substantial potential for use as antibacterials. For this reason, our interest has grown to extract SS from the cocoon of a very popular *B. mori* variety “Rajshahi Silk” and to introduce a green synthesis approach for the extraction of AgNPs in an aqueous neutral condition. The green synthesis of silver nanoparticles is primarily concerned with the selection of solvent medium, reducing agent, and nontoxic substances for stability [18, 19]. In this context, it is essential to mention that the synthesis of silver nanoparticles using biological systems makes nanoparticles more biocompatible and environmentally benign.

AgNPs have strong antibacterial action towards gram-positive, or gram-negative bacteria are reported in many past studies. Sondi and Salopek-Sondi first observed that AgNPs accumulate in the cellular membrane and form pits in the bacterial cell wall of *Escherichia coli* which led to an augmented permeability of the cell wall and ultimately the cell death [20]. Shrivastava et al. revealed that AgNPs create disorder in the integrity of the bacterial cell wall and membrane, supporting the permeability of the membrane and the leakage of the cell constituents, and eventually induce cell death [21]. Afterward, many advanced studies have been reported that DNA and protein of bacterial cell were destroyed by peroxidation, dephosphorylation, etc., reaction of Ag^+^ ions in aerobic conditions [21–25]. Even then, some antibacterial mechanisms of AgNPs on multidrug-resistant bacteria remain unknowable. Nowadays, a group of pathogens namely ESKAPE (i.e., *Enterococcus faecalis*, *Staphylococcus aureus*, *Klebsiella pneumoniae*, *Acinetobacter baumannii*, *Pseudomonas aeruginosa*, and *Enterobacter species*) have become cause of several important nosocomial infections and already resistant to the last-line of antibiotics [26–29]. Yet again, Szmolka and Nagy were shown the multidrug resistivity of *Escherichia coli* in non-clinical sources [30]. In our study, we used *Escherichia coli* MZ20, *Pseudomonas aeruginosa* MZ2F, and *Pseudomonas aeruginosa* MZ4A. Previously, Zulkar et al. have reported that all bacteria are multidrug-resistant, e.g., *Escherichia coli* MZ20 is resistant to penicillin G (P), tetracycline (TE), cotrimoxazole (COT), erythromycin (E), kanamycin (K), streptomycin (S), ciprofloxacin (CIP), ceftazidime (CAZ), nalidixic acid (NA), colistin (CL), ceftriaxone (CTR), doxycycline (DO), amoxicillin (AMX); *Pseudomonas aeruginosa* MZ2F is resistant to P-TE-COT-E-K-S-CAZ-NA-DO-AMX, and *Pseudomonas aeruginosa* MZ4A is resistant to P-TE-COT-E-K-S-CAZ-NA-CL-DO-AMX [31].

In the present work, silver nanoparticles were synthesized using a simple, effective, and ecofriendly method using silk sericin. Silk sericin solution was extracted from *B. mori* silk cocoons and used as a reducing and stabilizing agent. The synthesized silver nanoparticles (AgNPs) were characterized using UV-visible spectroscopy, Fourier-transform infrared-attenuated total reflection (FTIR-ATR) spectroscopy, X-ray diffraction (XRD), and transmission electron microscopy (TEM). Additionally, we explored the antibacterial activity of biosynthesized silver nanoparticles (AgNPs) against multidrug-resistant *Escherichia coli* MZ20, *Pseudomonas aeruginosa* MZ2F, and *Pseudomonas aeruginosa* MZ4A.

### Experimental

#### Materials

Silk sericin is a protein that is extracted from silkworm cocoons. Silkworm cocoons were collected from the *Bangladesh Sericulture Research & Training Institute*, *Rajshahi*, *6207.* Silver nitrate Anhydrous (extra pure) was brought from MOLYCHEM, India. All the analytical grade reagents used in this study were purchased from Merck, Germany.

*Escherichia coli* MZ20, *Pseudomonas aerugiosa* MZ2F, and *Pseudomononas aeruginosa* MZ4A were collected from the Department of Biotechnology and Genetic Engineering of our university. Bacterial strains were collected from different sources, such as hospital waste material and poultry litters. All bacterial strains were resistant to multiple drugs [31].

#### Methods

##### Extraction of silk sericin

The silkworm cocoons were cut into small pieces and boiled at 90°C in demineralized water for 90 min at a ratio of 1 part cocoon to 40 parts water. Silk fibroin was separated using polyester filter cloths followed by Whatman filter paper. The SS solution was refluxed to remove volatile matters and concentrated until the desired concentration was achieved. The prepared SS colloidal solution (60 ml obtained from 5g of cocoons) was frozen at −20°C in a deep freeze (BIO Memory 175L) for about 12 h. Then, the frozen SS was lyophilized in a freeze dryer (Yamato Freeze Dryer DC 401) for about 24 h until dry. Then, the percentage of the solid content of silk sericin was calculated and found to have an average value of 17wt% sericin with respect to the weight of the silk cocoons.

##### Synthesis of silk sericin-capped silver nanoparticles

Aqueous silver nitrate solution was prepared for AgNP synthesis. The lyophilized silk sericin was placed in a conical flask containing an aqueous AgNO_3_ solution. Two molar ratios of SS to AgNO_3_ were used: 1:6 and 1:8. The silver ions were reduced to silver nanoparticles (AgNPs) within a few minutes at 65°C with continuous stirring. The color of the solution changed from yellow to brown. The SS-AgNPs synthesized using different molar ratios were characterized using UV-visible spectroscopy, FTIR-ATR spectroscopy, XRD, and TEM measurements.

#### Measurements

##### UV-visible spectroscopy

Sericin and SS-AgNPs were characterized using UV-1700 predominantly for accurate quantification of sericin protein and SS-AgNPs. The instrument can automatically calculate sample concentrations based on a standard curve using the K factor method.

##### Fourier-transform infrared-attenuated total reflection spectroscopy

FTIR (ATR) spectra of the sericin and SS-AgNPs were measured using an FTIR-ATR Instrument manufactured by SHIMADZU (IRAffinity-1S) with a resolution of 4 cm^−1^ in the wavenumber range 700–4000 cm^−1^.

##### Transmission electron microscopy

The size and shape of SS and SS-AgNPs were determined using TEM (model number is JEM-2100 F JEOL Japan) with ultra-high regulation FETEM operating at 200 kV.

##### XRD measurement

The crystalline behavior of SS-AgNPs was examined using an X-ray diffractometer (Rigaku Ultima IV) operating at a voltage of 40 kV and a current of 40 mA with CuKα (*λ* = 1.5406 Å) radiation and a programmable divergent slit. The samples were scanned in the 2θ range 10°–70° with a scanning speed and step size of 3° min^−1^ and 0.02°, respectively.

##### Antimicrobial assessment

The well diffusion method [32, 33] was used to test the antibacterial activities of synthesized silk sericin-silver nanoparticles (SS-AgNPs) according to the National Committee for Clinical Laboratory Standards (NCCLS) [34]. Three selected multidrug-resistant bacterial isolates (*Escherichia coli* MZ20, *Pseudomonas aerugiosa* MZ2F, *Pseudomononas aeruginosa* MZ4A were grown in Luria–Bertani (LB) for 18–20 h. The bacterial inoculums were prepared by maintaining a turbidity of 0.5 McFarland standard (equal to 1.5×10^8^ colony-forming units (CFU)/ml). Mueller Hinton agar (MHA) plates (150-mm diameter) were prepared, and bacterial suspensions were spread over the surface. The wells (9-mm diameter) were made using a cork borer in MHA plates. Each well was loaded with 70 μl of different concentrations (10 μg/ml, 20 μg/ml, 30 μg/ml, 40 μg/ml, and 50 μg/ml) of SS-AgNPs. Plates were incubated at 37 °C for 24 h. The diameters of the zones of inhibition around the wells were measured in millimeters (mm). The experiment was performed in triplicate*.* Data were expressed as the mean ± standard deviation.

## Results

We developed a convenient method for the extraction of relatively pure sericin protein from *B. mori* silkworm cocoon. Maximum 17 w/w% of yellow crude sericin powder was extracted after freeze-drying. The chemical composition viz. protein, sugar, ash, and amino acid of the isolated sericin powder have been identified by using the standard chemical test methods.

### UV-visible spectroscopic analysis

**Fig. 1:**
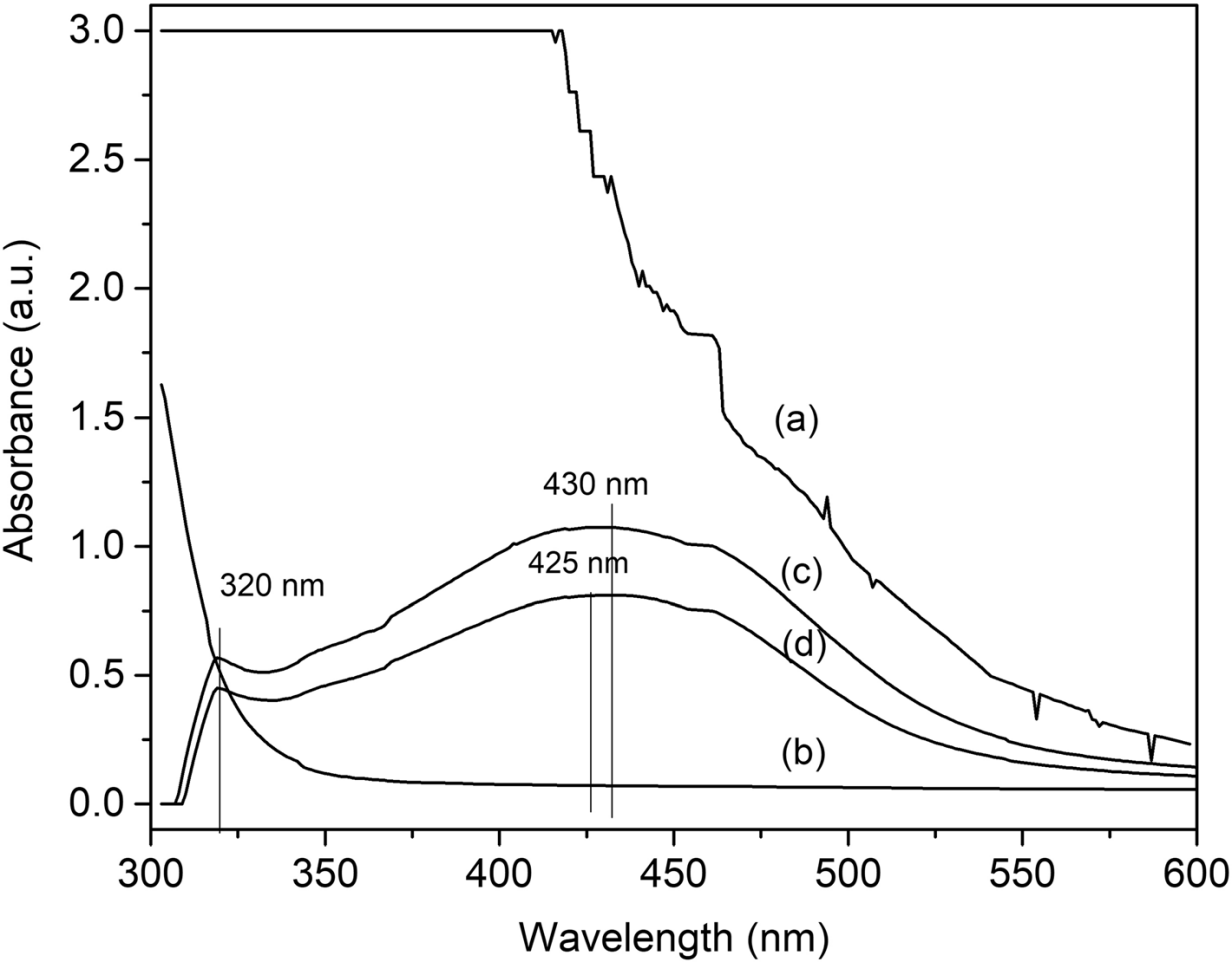
UV-visible absorption peak of **a** silk sericin (SS), **b** AgNO_3_, **c** 1:6 SS-AgNPs, and **d** 1:8 SS-AgNPs

UV-visible spectroscopy is a common technique used to characterize the formation of AgNPs. In our investigation, AgNPs were formed from silver nitrate through the reducing action of sericin, which was confirmed by the UV-visible spectra shown in Fig. 1. The absorption peak at about 425 nm (absorbance 0.810%) indicated the formation of AgNPs. The UV-visible spectrum of SS-AgNPs of different SS to AgNP ratios showed a similar trend. The results exhibited a broad absorbance peak centered at around 430 nm, which could be attributed to the surface plasmon resonance band of AgNPs [35]. In Fig. 1, the presence of AgNPs explained the observation of two surface plasmon resonance absorption bands at 320 and 430 nm. According to the theoretical calculation of Kelly et al. [36], the multiplicity was caused by the quadruple plasmon resonances and in-plane dipole of Ag nanoplates, respectively [36–38]. Thus, a small shoulder peak was found at 320 nm, implying the possible existence of different sizes and morphologies of AgNPs. The absorbance intensity of the 1:6 SS-AgNPs was higher than that of the 1:8 SS-AgNPs. The increasing silver fraction in the 1:8 SS-AgNPs could suggest that the silver ions were reduced continuously by sericin [16]. Another study mentioned that when the AgNO_3_ concentration was increased, tyrosine residues of sericin were gradually oxidized by silver ions, and reacting AgNO_3_ with SS to produce several AgNPs in solution was suggested [16].

### Fourier-transform infrared-attenuated total reflection spectroscopic analysis

**Fig. 2:**
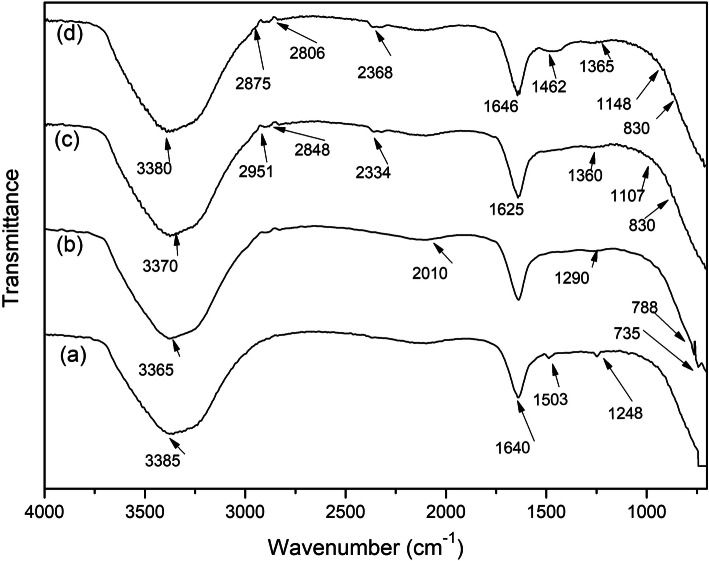
Fourier-transform infrared-attenuated total reflection spectra of **a** silk sericin, **b** AgNO_3_ solution, **c** 1:6 SS-AgNPs, and **d** 1:8 SS- AgNPs

The structure of SS, AgNO_3_, and SS-AgNP samples was characterized using FTIR-ATR spectroscopy. Figure 2 shows the original spectra of SS, _AgNO3_, and SS-AgNPs at different ratios. The typical absorption bands in sericin protein, such as Amide I (1700–1600 cm^−1^), Amide II (1560–1500 cm^−1^), and Amide III (1300–1200 cm^−1^) were detected at 1640, 1503, and 1248 cm^−1^, respectively, which are illustrated in Table 1. The absorption bands of 3 mM AgNO_3_ solution were detected at 735, 788, 1290, and 2010 cm^−1^ [39].

**Table 1:** Infrared band assignment of silk sericin (SS), AgNO_3_, and SS-Ag nanoparticles

Infrared (IR) band (cm^-1^)	Band assignment
1700–1600	Amide I (C=O stretching vibration)
1560–1500	Amide II (N–H bending and C–N stretching vibration)
1300–1200	Amide III (C–N–H in plane bending and C–N stretching vibration)
1107,1148, 1360,1462	Free carboxylate groups (COO– stretching vibration)
3365, 3370, 3380, 3385	Amine N–H stretching vibration
830	Amine salt

Absorption bands of the 1:6 SS-AgNPs showed new functional groups, including carboxylate (1107 and 1360 cm^−1^) and amine salt (830 cm^−1^) and the absorption bands of the 1:8 SS-AgNPs showed the same functional groups as the 1:6 SS-AgNPs, including carboxylate (1148, 1365, and 1462 cm^−1^) and amine salt (830 cm^−1^). The peak at 1248 cm^−1^ could be assigned to the phenolic group of sericin, and the disappearance of the peak 1248 cm^−1^ at SS-AgNPs was due to the oxidation of the carboxyl group peak, which was identified at 1360, 1365, and 1462 cm^−1^ in Fig. 2. The SS-AgNPs represented the peak of Amide I at 1625 and 1640 cm^−1^ and amine N–H stretching vibration at 3365–3385 cm^−1^ at 1:6 and 1:8 ratios, respectively, and the formation of AgNPs did not significantly alter the structure of sericin [11].

### Transmission electron microscopy

**Fig. 3:**
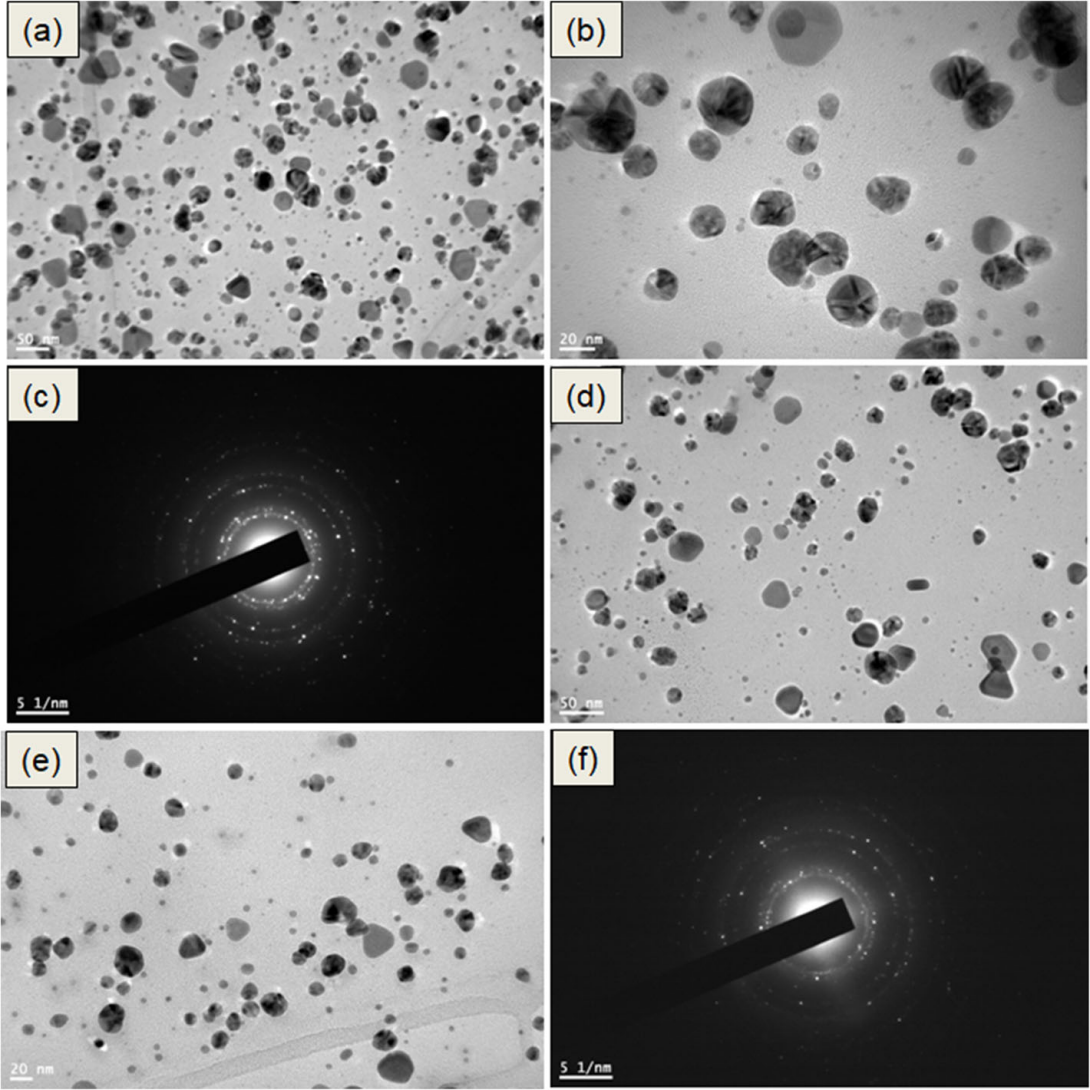
Transmission electron microscopy images of synthesized silver nanoparticles—1:6 SS-AgNPs (**a**–**c**) and 1:8 SS-AgNPs (**d**–**f**)

TEM was conducted to determine the size and shape of nanoparticles. Figure 3a–e shows the TEM image of the 1:6 SS-AgNPs and 1:8 SS-AgNPs, respectively. It was observed from the images that the synthesized SS-AgNPs at both the 1:6 and 1:8 mole ratios dispersed well in the aqueous solution. Most of the synthesized SS-AgNPs were clearly observed in the size range of 5–25 nm in Fig. 3b, e. The average particle size of the 1:6 SS-AgNPs was 10–30 nm and that of the 1:8 SS-AgNPs was 5–20 nm. The high resolution of TEM images showed the lattice structure of AgNPs with a range of 0.25 nm, which was indicated by the face-centered cubic crystalline structure of the metallic AgNP plane of cubic silver [8, 40].

### X-ray diffraction analysis

**Fig. 4:**
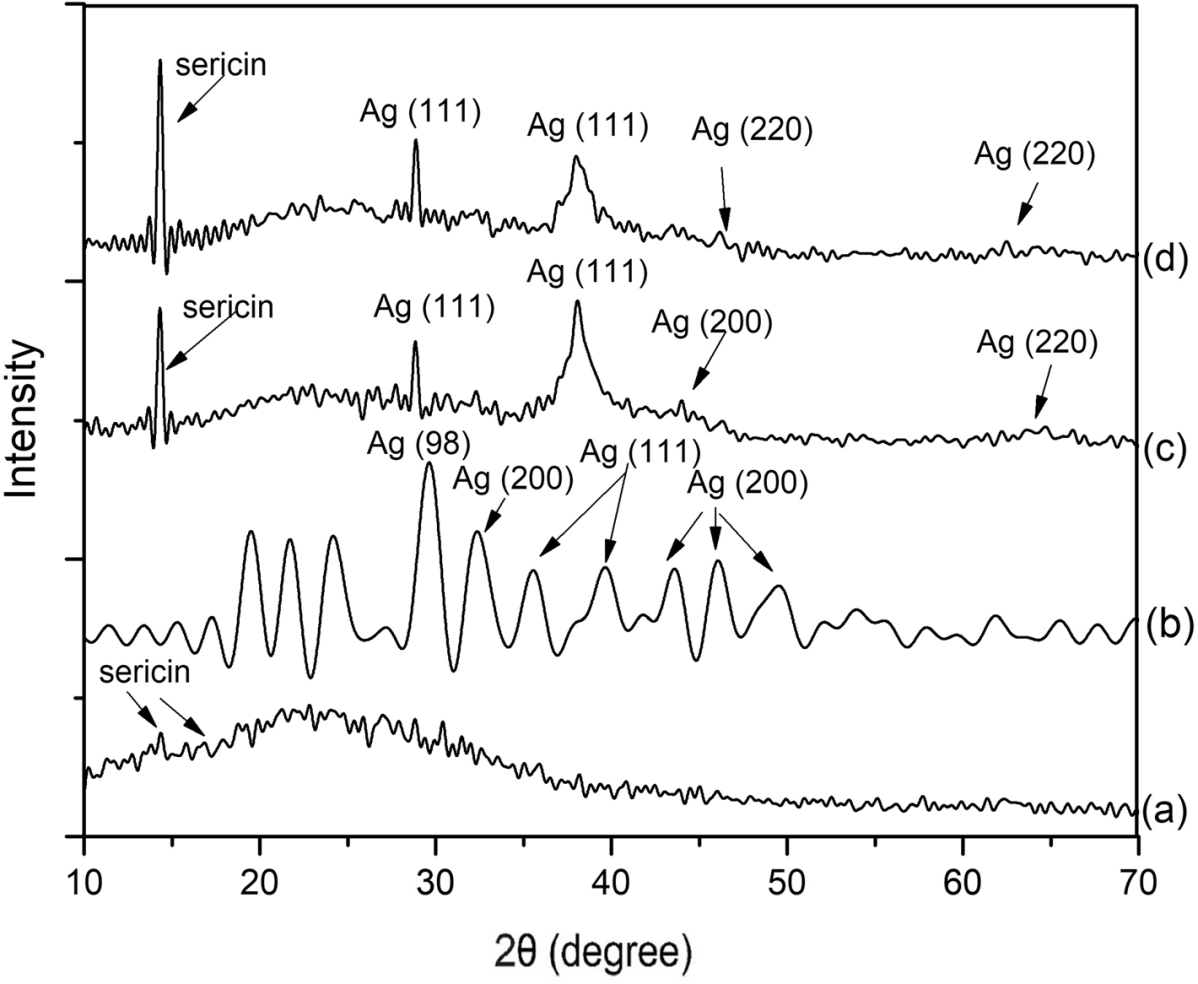
X-ray diffraction diagram of **a** silk sericin, **b** AgNO_3_ solution, **c** 1:6 SS-AgNPs, and **d** 1:8 SS-AgNPs

Figure 4 shows the crystalline structure of SS, AgNO_3_, and SS-AgNPs obtained using XRD (Fig. 4). The XRD spectrum of SS, AgNO_3_, and SS-AgNPs has shown the characteristic patterns of the different crystalline structures, which are summarized in Table 2. The value of 2θ at 14.9° and 19.2° could be related to the crystalline and amorphous organic phase of SS. Silver nitrate showed a cubic phase of Ag_2_O crystal and a face-centered cubic crystalline structure of metallic Ag. It was detected at 29.5°, 32.3°, 44.3°, and 48.8°, which corresponds to the (98), (200), (200), and (200) plane of cubic silver and 38.4° and 38.8° indicated (111), (111), the face-centered cubic crystalline structure of metallic Ag.

**Table 2:** X-ray diffraction scattering data for various crystalline structures

2*θ*	Plane	Structure
64.4, 46.3	220	Face-centered cubic structure of metallic AgNPs
32.3, 44.3, 48.8	200	Cubic phase of Ag_2_O crystal
38.2, 38.4, 38.8	111	Face-centered cubic crystalline structure of metallic AgNPs
27.9, 28.2	111	Cubic phase of Ag_2_O crystal
29.5	98	Pure silver crystal
14.9, 19.2		Sericin crystalline and amorphous organic phase

Again, the X-ray diffractogram made evident that the AgNPs were formed by the reduction of Ag^+^ ions to Ag^0^ by the SS extract. The characteristic diffraction peaks at scattering angles (2θ) of about 38.2°, 44.3°, and 64.4°, which were attributed to the (111), (200), and (220) planes of the silver, respectively. Previous studies have also affirmed that the resultant particles are face-centered cubic structures of metallic AgNPs. The other peaks observed at 27.9°, 32.3°, and 46.3° were assigned to the (111), (200), and (220) planes of the cubic-phase Ag_2_O crystals in the sample [41]. This finding clearly indicates that the silver nanoparticles formed by the reduction of Ag^+^ to Ag^0^ by the redox-active nature of tyrosine residues are crystalline in nature [42].

## Discussion

**Fig. 5:**
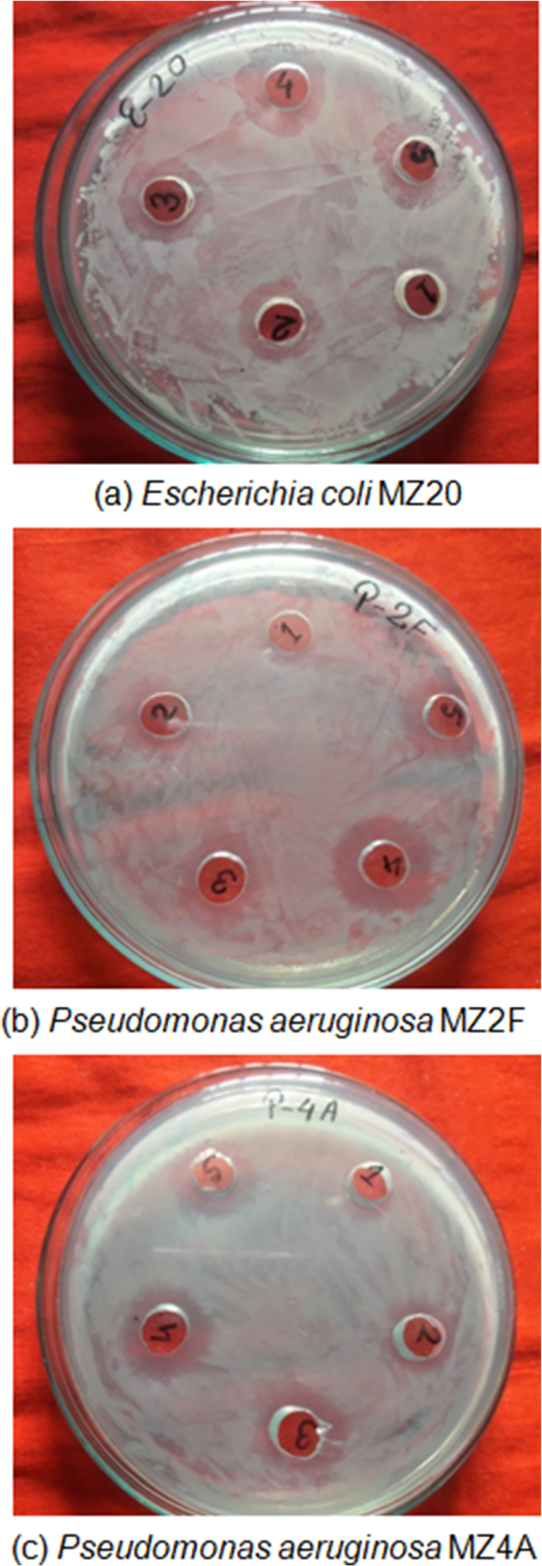
Antibacterial activities of different concentrations of silk sericin-silver nanoparticles on multidrug-resistant *Escerichia coli* and *Pseudomonas aeruginosa*

**Fig. 6:**
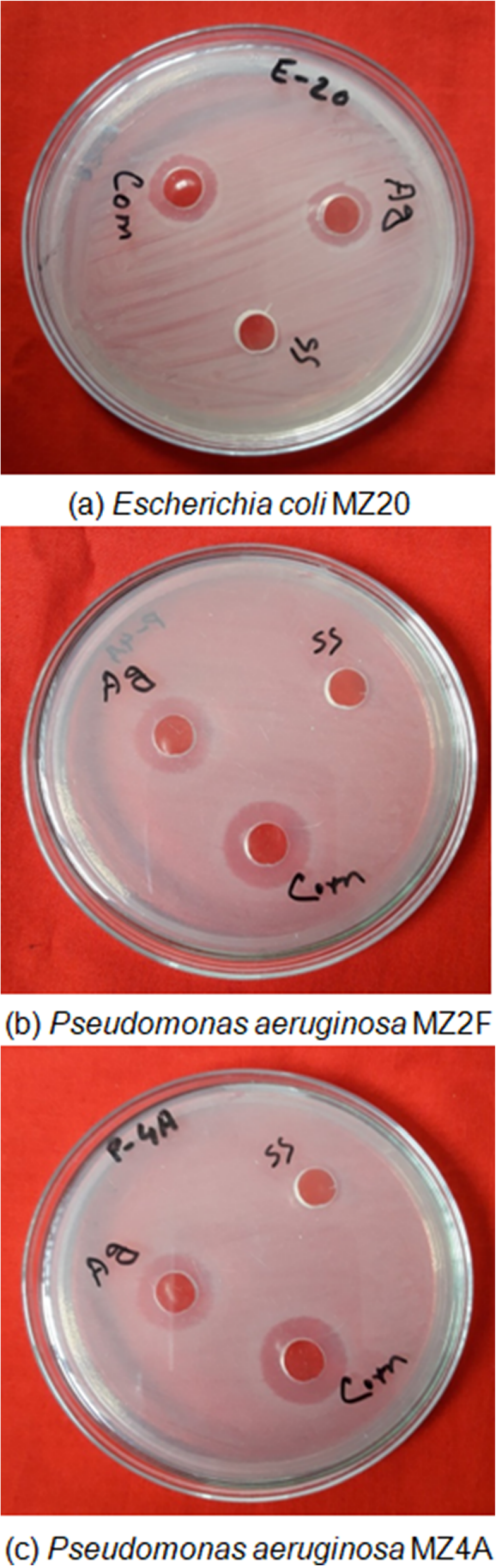
Antibacterial activities of silk sericin (SS), 3 mM of silver nitrate (Ag), and SS-AgNPs (Com) on multidrug-resistant *Escerichia coli* and *Pseudomonas aeruginosa*

The emergence of multidrug-resistant microorganisms is a serious concern for the medical sector, as it is difficult to control them with available antibiotics. So, it is essential to find out an alternative antimicrobial agent to control these multidrug-resistant microorganisms. Ecofriendly synthesized SS-AgNPs could be a potential candidate to control them. SS-AgNPs were synthesized and their antibacterial activity was investigated against drug-resistant pathogenic microorganisms, such as *Pseudomonas aeruginosa* and *Escherichia coli*. Tested pathogens (*Pseudomonas aeruginosa* and *Escherichia coli)* are resistant to different antibiotics reported [31]. The results show that eco-friendly synthesized SS-AgNPs have strong antibacterial efficacy against tested multidrug-resistant pathogens such as *E. coli* and *P. aeruginosa* (Fig. 5). Figure 5 reveals the inhibition zones around the well treated with SS-AgNPs. The antibacterial activity against pathogens was measured by calculating the diameter of the inhibition zone. The average results of the zone of inhibition are shown in Table 3. The MIC and MBC of SS-AgNPs against *E. coli* and *P. aeruginosa* were also determined. Figure 5 and Table 3 reveal that synthesized SS-AgNPs showed MIC of 20 μg/mL against both *E. coli* and *P. aeruginosa*. We compared the antibacterial activity of SS-AgNPs with SS and 3 mM of AgNO_3_. The SS-AgNPs synthesized from 1:6 Silk sericin and 3 mM of AgNO_3_ were selected for the study due to their high yield and stability. It has been proved that the cellular membrane of bacteria has a negative charge due to the presence of carboxyl, phosphate, and amino groups [43]. The positive charge confers electrostatic attraction between AgNPs and negatively charged cell membrane of the microorganisms, thereby facilitates AgNP attachment onto cell membranes [44]. It has also been reported that AgNPs can anchor to the bacterial cell wall and consequently infiltrate it. This action will cause physical changes in the bacterial membrane, like the membrane damage, which can lead to cellular contents leakage and bacterial death [45, 46]. Furthermore, the antibacterial mechanism of AgNPs is also due to their ability of producing high levels of reactive oxygen species (ROS) and free radical species which inhibited respiration and growth of cells [47, 48]. It is clearly observed in Fig. 6 that silk sericin did not have any activity against multidrug-resistant bacterial strains. However, 3 mM of AgNO_3_ solution and SS-AgNPs showed significant inhibition zones against tested drug-resistant *E. coli* and *P. aeruginosa* (Fig. 6 and Table 4). The results suggest that SS-AgNPs could be useful as an antimicrobial agent to control drug-resistant pathogens. The results of this study are consistent with previously reported studies [49, 50].

**Table 3:** Antibacterial activity of synthesized silk sericin-capped silver nanoparticles (SS-AgNPs) against multidrug-resistant *Escerichia coli* and *Pseudomonas aeruginosa*

Tested bacteria	Zone of inhibition (mm)				
	SS-AgNPs (μg/ml)				
	10	20	30	40	50
*Escherichia coli* MZ20	×	15 ± 0.40	17 ± 0.38	18.5 ± 0.34	20 ± 0.41
*Pseudomonas aeruginosa* MZ2F	×	16 ± 0.18	16.5 ± 0.36	19 ± 0.20	18.5 ± 0.70
*Pseudomonas aeruginosa* MZ4A	×	14.5 ± 0.2	17 ± 0.18	18 ± 0.31	18 ± 0.40

**Table 4:** Antibacterial activity of silk sericin, AgNO_3_, and 1:6 SS-AgNPs against multidrug-resistant *Escerichia coli* and *Pseudomonas aeruginosa*

Tested bacteria	Zone of inhibition (mm)		
	Silk Sericin (SS) 50 μg/ml	3 mM Silver nitrate 50 μg/ml	1:6 SS-AgNPs composite 50 μg/ml
*Escherichia coli* MZ20	×	16 ± 0.92	20 ± 0.41
*Pseudomonas aeruginosa* MZ2F	×	17 ± 0.43	18.5 ± 0.70
*Pseudomonas aeruginosa* MZ4A	×	17 ± 0.66	18 ± 0.40

**Antimicrobial activity of synthesized SS-AgNPs**

## Conclusions

The aim of this study was to recover SS from the wastewater of the silk degumming process, which causes water pollution by increasing BOD and COD. In this research, dry SS (17 w/w% of cocoon) was recovered by freeze-drying without adding any reagent. Silver nanoparticles were synthesized and stabilized by extracted SS using a green synthesis technique. The SS-AgNPs were characterized using UV-visible spectroscopy, FTIR-ATR spectroscopy, TEM, and XRD measurement. The antibacterial activity of SS-AgNPs was examined. The results of the study are summarized briefly below:

- UV-visible spectroscopic absorbance showed a high percentage of SS-AgNPs was formed at a higher concentration of silver nitrate solution.
- The primary and secondary amide (Amide I, Amide II) absorption peak position of SS changed after the synthesis of SS-AgNPs.
- The remarkable crystalline structure of SS-AgNPs was shown in the X-ray diffractogram, which was different from that of the SS and silver nitrate crystalline structures.
- The sizes of SS-AgNPs were ~20–120 nm, which was observed by TEM.
- SS-AgNPs showed a substantial antibacterial effect against multidrug-resistant *E. coli* and *Pseudomonas aeruginosa.*

Therefore, it can be concluded that the green method of SS-AgNP synthesis is very simple, economical and ecofriendly and reduces the use of toxic and pollution-causing reducing agents and stabilizers. Since the SS-AgNPs have good antimicrobial properties, they could be used for biomedical purposes, especially in wound dressing. Future research should focus on the application of SS-AgNPs in membrane filters and hydrogels.

## Data Availability

Not applicable.

## Abbreviations

BOD: Biological oxygen demand
COD: Chemical oxygen demand
FETEM: Field emission transmission electron microscopy
FTIR-ATR: Fourier-transform infrared-attenuated total reflection
LB: Luria–Bertani
NCCLS: National Committee for Clinical Laboratory Standards
SS: Silk sericin
SS-AgNPs: SS-capped silver nanoparticles
TEM: Transmission electron microscopy
XRD: X-ray diffraction
